# The Contributions of Informal Home Literacy Activities to Specific Higher-Level Comprehension Processes

**DOI:** 10.11114/jets.v6i12.3627

**Published:** 2018-12

**Authors:** Brenda Ann Marie Hannon

**Keywords:** home literacy environment, comprehension processes, word skills, preschoolers

## Abstract

This study shows that home literacy activities contribute to kindergarten children’s higher-level comprehension processes, namely knowledge integration and knowledge access. Kindergarten children completed measures assessing literacy and language skills and then their performances on these measures were correlated with home literacy activities, which were assessed via a parental questionnaire. Consistent with previous research, the results revealed that informal home literacy activities were positively related to language comprehension and vocabulary but not to letter-word decoding and phonemic decoding skills. The results also revealed that home literacy activities were positively related to knowledge integration and knowledge access, two strong predictors of language and reading comprehension. Finally, the present results suggest that the contributions that home literacy activities make to language comprehension are the same contributions that home literacy activities make to higher-level comprehension processes. In other words, the contributions that home literacy activities make to language comprehension are not independent of the contributions that home literacy activities make to higher-level comprehension processes.

## 1. Introduction

### 1.1 Introduction to Problem

Most theoretical models of reading depict reading as a complex ability that requires the execution of many cognitive components. Word decoding skills are used to both orthographically and phonemically decode letters and identify meanings of words; higher-level knowledge integration processes access and integrate prior knowledge with new explicit and implicit information from a text; and planning/organization skills organize active information in a reader’s working memory so that a mental representation of a text can be constructed (Hannon, 2012; Hannon, 2013). These cognitive components not only begin to develop at an early age but are also highly predictive of preschoolers’ language comprehension abilities (e.g., Hannon & Frias, 2012), beginning reading (e.g., August, Francis, Hsu, & Snow, 2006; Cain, Oakhill, & Bryant, 2004), and adult reading (e.g., Hannon & Daneman, 2001; Hannon & Daneman, 2006; Hannon & Daneman, 2009; Hannon & Daneman, 2014). In addition, a growing body of research continues to document the contributions of the home literacy environment to children’s early literacy skills, including those contributions to the development of language comprehension (Sénéchal & LeFevre, 2002), vocabulary (e.g., Niklas & Schneider, 2013), phonemic awareness (e.g., Foy & Mann, 2003), and alphabet knowledge (e.g., Niklas & Schneider, 2013; Sénéchal & LeFevre, 2002; Sénéchal & LeFevre, 2014). However to date no study has examined the contributions of the home literacy environment to comprehension processes (e.g., text processing, knowledge integration) even though these processes are strong predictors of preschoolers’ language comprehension (e.g., Hannon & Frias, 2012) and early reading (e.g., August et al., 2006; Cain et al., 2004). Therefore, the major objective of this paper is to determine whether the home literacy environment contributes to the development of specific higher-level comprehension processes; see Endnote 1. Below I briefly review the literature about home literacy activities and their contributions to early literacy and language skills a as well as the literature about specific higher-level comprehension processes; see Endnote 2. In the last section I describe the present study.

### 1.2 Home Literacy Environment

Considerable research has documented the importance of the home literacy environment as a precursor to a number of early literacy and language skills (Blewitt, Rump, Shealy, & Cook, 2009; Chow, McBride, Cheng, Cheung, & Chow, 2008; Weigel, Martin, & Bennett, 2006). For instance home literacy activities, such as parent-child shared storybook reading and home characteristics, such as the education level of the parents, have been related to preschoolers’ language comprehension (e.g., Sénéchal & LeFevre, 2002), phonemic awareness (e.g., Foy & Mann, 2003), alphabetic knowledge (e.g., Niklas & Schneider, 2013, Sénéchal & LeFevre, 2002; Sénéchal & LeFevre, 2014), vocabulary knowledge (e.g., Blewitt et al., 2009; Sénéchal & LeFevre, 2002; Sénéchal & LeFevre, 2014), morphological knowledge (e.g., Chow et al., 2008), and print knowledge (e.g., Weigel et al., 2006). These home activities/characteristics are also related to grade 1 children’s reading ability (e.g., Niklas & Schneider, 2013; Sénéchal, 2006), vocabulary knowledge (e.g., Niklas & Schneider, 2013; Sénéchal, 2006) and phonemic awareness (e.g., Sénéchal, 2006). Moreover, home literacy activities are related to a child’s level of engagement in literacy activities (Mascarenhas et al., 2017), regardless of the language spoken at home (e.g., Farver, Xu, Lonigan, & Eppe, 2013). Finally a recent study targeting English-speaking children who were schooled in French, suggests that the contributions of home literacy activities to early literacy and language skills are independent of early schooling (i.e., kindergarten and grade 1; see Sénéchal & LeFevre, 2014). This latter finding provides strong support that the home literacy environment is a unique contributor to the development of children’s literacy and language skills because they are independent of language skills and formal schooling they are independent of language skills and formal schooling.

Much of the early home literacy research conceptualized the home literacy environment as uni-dimensional (see Burgess, Hecht, & Lonigan, 2002; Weigel et al., 2006 for discussions and examples). This research typically queried parents about a number of home literacy activities and then correlated parents’ responses to children’s literacy and language skills. More recent research, however, conceptualizes the home literacy environment as multi-dimensional. The *Home Literacy Model*, a model developed by Sénéchal and colleagues, for example, separates home literacy activities into two dimensions--formal literacy activities and informal literacy activities--and each dimension of activities makes a separate and independent contribution to the development of children’s literacy and language skills (e.g., Sénéchal & LeFevre, 2002; Sénéchal & LeFevre, 2014).

According to the Home Literacy Model, formal literacy activities focus on the print itself; for example, teaching a child how a letter or word sounds (Sénéchal, 2006). As noted by Sénéchal (2006), the term *formal* is used to describe the approach used to teach a child rather than the structure of the materials. For example, parents adopting an educational/teacher role will teach their child about the sounds of letters. On the other hand, a workbook that includes a structured activity about the alphabet or phonological sounds is not a formal activity. Examples of formal activities include parents teaching their child the names of letters, how to sound out letters and/or words, or how to print his/her name.

Like formal activities, informal home literacy activities in the Home Literacy Model also include print; however, the primary focus of informal home literacy activities is *not* the print. Nor do parents adopt a “teaching focus.” Rather, the primary focus of informal literacy activities is the child’s understanding of the story. Because of the nature of this focus, the contributions of informal home literacy activities to the development of literacy skills is incidental inasmuch as the development of literacy skills is a consequence of shared parent-child shared storybook reading rather than the actual teaching of specific literacy skills (Sénéchal, 2006). Examples of informal activities include frequency of parent-child shared storybook reading, number of questions asked during parent-child shared storybook readings, and how long parents have been reading to their child.

The Home Literacy Model has considerable support in the literature with some of the most cogent research showing independence between measures of formal and informal home literacy activities. For example, Sénéchal and LeFevre (2014) showed that a formal literacy activity was as predictive of alphabet knowledge (i.e., *r* = .33) but not receptive vocabulary (i.e., *r* = .08). In contrast, an informal literacy activity, such as parent-child shared storybook reading, was predictive of receptive vocabulary (i.e., *r* = .31) but not alphabet knowledge (i.e., *r* = .15). Similarly, Sénéchal and LeFevre (2002) showed that formal home literacy activities were predictive of phonemic awareness (i.e., *r* = .36) and alphabet knowledge (i.e., *r* = .21) but not vocabulary knowledge (i.e., *r* = .15). In contrast, informal home literacy activities were predictive of language comprehension (i.e., *r* = .31) and vocabulary knowledge (i.e., *r* = .38) but not phonemic awareness (i.e., *r* = .10) and alphabet knowledge (*r* = .08).

Nevertheless, absent from this research is an examination of the contributions of the home literacy environment to specific higher-level comprehension processes, such as memory for new facts, memory for implicit facts, and knowledge integration (i.e., the integration of prior knowledge with new text-based information). The absence of this research is problematic because specific higher-level comprehension processes are important contributors to early language comprehension abilities (Hannon & Frias, 2012).

### 1.3 The Relationships between Higher-Level Comprehension Processes and Language Comprehension

As noted in the introduction, most theoretical models depict reading and language comprehension as complex cognitive abilities that tap a wide range of cognitive component skills. Some of the most, if not the most, important, components are higher-level comprehension processes-- characterized as those processes that: (i) connect or *bridge* ideas in a text, (ii) infer meanings of words or themes from a text, (iii) identify the antecedent referents of pronouns, and (iv) predict future outcomes of a text— because these cognitive components are strong predictors of early language comprehension (August et al., 2006; Hannon & Frias, 2012). For example, Hannon and Frias (2012) showed that multiple measures of specific higher-level comprehension processes, namely knowledge integration, text memory, and the ability to access prior knowledge, accounted for 49.3% of the variance in prereaders’ language comprehension ability. Similarly, August et al. (2006) showed that higher-level comprehension processes accounted for a considerable amount of in reading and language comprehension in beginning readers. In addition, higher-level comprehension processes, such as those processes for learning and integrating text, are the principal processes for constructing a coherent mental representation of a text (Kendeou, van dan Broek, White, & Lynch, 2009).

With respect to the Home Literacy Model, given that higher-level comprehension processes account for large portion of variance in language comprehension (Hannon & Frias, 2012) and given that informal home literacy activities contribute to language comprehension (Sénéchal & LeFevre, 2002), it is plausible that measures of informal home literacy activities will also contribute to specific higher-level comprehension processes. That is, given that specific higher-level comprehension processes → language comprehension and given that informal home literacy activities -> language comprehension, it is possible that informal home literacy activities → specific higher-level comprehension processes. Because of this possibility, the present study hypothesizes that informal home literacy activities contribute to higher-level comprehension processes.

### 1.4 Current Study

Although the contributions of the home learning environment to the precursors of literacy acquisition have been researched for nearly three decades, based on a literature review this research has yet to investigate the contributions of the home literacy environment to some of the most important predictors of reading and language comprehension, namely specific higher-level comprehension processes. Therefore this study examines the contributions that informal home literacy activities, such as the number of books that are read to a child and the number of questions that are asked per book, make to the higher-level comprehension processes (e.g., knowledge integration, text memory, text inferencing, and knowledge access) of preschool children. Finally, measures of letter-word decoding and phonemic awareness were included in order to confirm discriminant validity of the informal home literacy activities. That is, given that informal home literacy activities are not predictive of letter-word decoding and phonemic awareness (e.g., Sénéchal & LeFevre, 2002; Sénéchal & LeFevre, 2014), measures of letter-word decoding and phonemic awareness were included here in order to show that measures of informal home literacy activities do not correlate with all literacy measures.

The measure of specific higher-level comprehension processes was Hannon and Frias’s (2012) prereader component processes task. This task is unique inasmuch as it provides estimates of separate higher-level comprehension processes: text memory, text inferencing, knowledge integration, and knowledge access. Moreover, it accounts for as much as 49% of the variance in preschoolers’ comprehension ability. Finally, the measure of language comprehension was an age-appropriate standardized measure, called the Gates McGinitie (e.g., MacGinitie & MacGinitie, 1989) and the word decoding measures were the letter-word identification and word attack subtests of the widely used Woodcock Reading Mastery Test-Revised (e.g., Woodcock, 1987).

## 2. Method

### 2.1 Participants

One hundred and twenty children (49 girls, 71 boys) were tested in kindergarten, *M* age = 5.75 years. These native English-speaking children were recruited from local schools and libraries. Eighty-seven were of Hispanic descent and 33 were of non-Hispanic descent. Reports from parents and teachers indicated that all of children were free of learning and/or psychological disabilities. Children received a toy package for participating.

The education level of parents was a control variable. This control variable was calculated by averaging the mother’s and father’s education. In instances of single-family households the education level of the single parent was used. The education level of the parents was average, with 11% completing high school, 59.2% completing some community college after high school, and 30% completing a university degree.

### 2.2 Measures

#### 2.2.1 Home Literacy Experiences

Two open-ended questions were used to assess informal home literacy experiences. First, parents were asked how many storybooks they read with their child each week, regardless of the time of day. On average, 4.4 books were read each week, *SD* = 3.61 books. For the purposes of this study this dependent variable is called books per week. Second, parents were asked the number of questions they asked per book. On average, 1.82 questions were asked per book, *SD* = 1.00 questions. For the purposes of this study this dependent variable is called number of questions per book. A composite z-score of informal home literacy activities was calculated using parent’s responses to these two questions. Formal literacy activities were not assessed.

#### 2.2.2 Measure of Language Comprehension

The standardized measure of language comprehension was the PR version of the Gates MaccGinitie, which is suitable for kindergarten children (MacGinitie & MacGinitie, 1989). This measure is used by many schools systems (e.g., MacGinitie & MacGinitie, 1989) and includes four short passages that are further divided into five segments each. The researcher reads aloud a passage segment and its accompanying question and the child selects which picture, from among three choices, answers the question correctly. There are 20 questions in total. The Cronbach alpha for this measure was .77.

#### 2.2.3 Letter-Word Decoding and Phonemic Awareness

Letter-word decoding skills and phonemic awareness were assessed using the letter-word identification and letter-word attack subtests of the Woodcock Reading Mastery Test-Revised (Woodcock, 1987); both subtests are suitable for children as young as two years old. Each subtest becomes progressively more difficult with each successive item and is discontinued after four consecutive wrong answers. Examples of stimuli from the letter-word identification subtest are: *m, d, cat, dog, then* and examples of stimuli from the letter-word attack subtest are: *k, n, nat, ep, tiff*. The Cronbach alphas for these subtests were .95 and .93 respectively.

#### 2.2.4 Vocabulary Knowledge

Vocabulary knowledge was assessed using the Assessment of Literacy and Language (i.e., ALL; Lombardino, Lieberman, & Brown, 2005), a 20-item picture-based receptive vocabulary measure. Each successive item increases in difficulty and administration is discontinued after six consecutive incorrect answers. Each item includes a sentence and four pictures. The child’s task is to identify the correct picture for a word. The Cronbach alpha for this measure was .74.

#### 2.2.5 Higher-Level Comprehension Processes

Specific higher-level comprehension processes were assessed using Hannon and Frias’s (2012) preschooler component processes task (PR-CPT). Because the PR-CPT is documented well in Hannon and Frias, it is only briefly described here. The PR-CPT includes 5 paragraphs; see Table 1 for a complete example. Each paragraph includes three parts that appear in the following order: (1) an animated introduction, (2) a paragraph, and (3) audio test statements assessing different higher-level comprehension processes.

An *animated introduction* provides a contextual framework for a paragraph. After the animated introduction, children listen to a two-sentence auditory paragraph while viewing its two accompanying pictures. The paragraph describes the semantic relationships among real (e.g., *shark*) and nonsense terms (e.g., *LORK, PARM*) that are depicted in the pictures. Following the paragraph, children answer Yes/No test statements assessing different comprehension processes; half of the statements are true and half are false. *Text memory* statements assess memory for explicit information presented in a paragraph; no prior knowledge is required (e.g., *A LORK looks like a SHARK*). *Text inferencing statements* assess memory for implied information in a paragraph; no prior knowledge is required (e.g., *A PARM looks like a SHARK*). *Knowledge access* statements assess the ability to access facts from prior knowledge; no visual or aural text-based information is required (e.g., *A TURTLE is smaller than a SHARK*). Finally, *knowledge integration* statements assess the ability to access facts from prior knowledge and integrate these facts with new text-based information. (e.g., *A LORK is smaller than a WHALE)*. There are three types of knowledge integration statements that vary in their use of abstract information.

In total, children completed 24 text memory statements, 8 text inferencing statements, 4 knowledge access statements, and 24 knowledge integration statements. In order to make the results easier to interpret, composite z-scores were calculated for text processing (text memory, text inferencing), and knowledge integration (low knowledge integration, medium knowledge integration, and high knowledge integration). The Cronbach alphas for the test statements ranged from .69 to .73.

## 3. Results

### 3.1 Descriptive Statistics

The descriptive statistics for all of the measures are reported in Table 2. As Table 2 shows, all the measures had wide ranges, which suggests large individual differences. It should be noted that this observation also applied to the competent measure for the home learning activities, which ranged from the 1^st^ percentile (i.e., z = −3.05) to the 99^th^ percentile (i.e., z = 5.75). In addition, the skews and kurtosis were all < 3.0, which suggests normal distributions. There was no missing data.

### 3.2 Correlations

Table 3 shows the correlations. Consistent with Hannon and Frias (2012), language comprehension correlated more strongly with the composite measures of text processing and knowledge integration than it did with word decoding, phonemic awareness, and knowledge access, *r’s* = .61 and .71 versus *r’s* = .42, .34, and .43 respectively.

According to the Home Literacy Model, the composite measure of informal home literacy activities should be significantly related to the measures of language comprehension and vocabulary knowledge but not measures of letter-word decoding and phonemic awareness. As Table 3 shows, there was good support for the Home Literacy Model. That is, consistent with the Home Literacy Model, the composite measure of informal home literacy activities was correlated with measures of language comprehension and vocabulary knowledge but not measures of letter-word decoding and phonemic awareness, *r* = .20 and *r =* .21 versus *r’s* = .09 respectively.

With respect to specific higher-level comprehension processes, given that the Home literacy Model suggests that informal home literacy activities contribute to language comprehension (Sénéchal & LeFevre, 2002) and given that developmental research suggests that higher-level comprehension processes (e.g., text-based processing, knowledge integration, knowledge access) account for a large portion of variance in preschoolers’ language comprehension abilities (Hannon & Frias, 2012), the present study hypothesized that the composite measure of informal home literacy activities should also contribute to higher-level comprehension processes. As Table 3 shows, consistent with this hypothesis the composite measure of informal home literacy activities correlated significantly with the knowledge integration and knowledge access *r’s* = .25 and .31 respectively. However, inconsistent with this proposal the composite measure of informal home literacy activities did not correlate significantly with text processing, *r* = .14. As Table 4 shows, this latter pattern of significant and non-significant correlations was also true for the partial correlations between the composite measure of informal home literacy activities and text processing, knowledge integration, and knowledge access after the influences of parental education were removed. This finding suggests that home literacy activities are more related to a child’s abilities to retrieve information from his/her prior knowledge and integrate this prior knowledge with new text-based information than they are with learning new information.

### 3.3 Regression Analyses

In order to examine the contributions that home literacy activities make to language comprehension and specific higher-level comprehension processes, two regression models predicting language comprehension were completed. The first regression entered the composite measure of informal home literacy activities into the model first and the measures of higher-level comprehension processes second. The second regression entered the measures of higher-level comprehension processes into the model first and the composite measure of informal home literacy activities second. The logic here is that if informal home literacy activities independently contribute to both language comprehension and higher-level comprehension processes (i.e., home literacy activities → language comprehension and home literacy activities → higher-level comprehension processes) than the composite measure of informal home literacy activities should account for a significant amount of variance in both regression models, regardless of the order that the composite measure of informal home literacy activities is entered into the models. Such findings also support earlier research that suggests that informal home literacy activities make direct contributions to language comprehension (e.g., Sénéchal & LeFevre, 2002). On the other hand, if the contributions that informal home literacy activities make to language comprehension are not independent of the contributions that informal home literacy activities make to higher-level comprehension processes than the composite measure of informal home literacy activities should account for a significant amount of variance in language comprehension when entered into the regression model first but it should not account for a significant amount of variance in language comprehension when it is entered into the regression model last. Such findings, if true, suggest that the shared variance between informal home literacy activities and language comprehension is the same shared variance that is between informal home literacy activities and higher-level comprehension processes. Table 5 shows the results of the two regressions.

As Table 5 shows, when the composite measure of informal home literacy activities was entered into the regression first, it accounted for 4.0% of the variance in language comprehension performance and the measures of the higher-level comprehension processes accounted for an additional 51.6% variance when they were entered second. However, when the composite measure of informal home literacy activities was entered into the regression second and the measures of the higher-level comprehension processes first, the composite measure of informal home literacy activities accounted for 0% of the variance in language comprehension performance. These findings suggest that the shared variance between the measures of home literacy activities and language comprehension is the same shared variance between the measures of home literacy activities and higher-level comprehension processes. In other words, the contributions that home literacy activities make to language comprehension are not independent of the contributions that home literacy activities make to higher-level comprehension processes.

## 4. Discussion

This study extended the Home Literacy Model in three important ways. First, the results showed that a composite measure of informal home literacy activities was positively related to measures of specific higher-level comprehension processes, which are strong predictors of reading and language comprehension. Second, the results showed that the contributions that informal home literacy activities make to language comprehension perfectly overlap with the contributions that informal home literacy activities make to higher-level comprehension processes. Third, the present results potentially links the Home literacy Model to a frequently used model of reading, namely the Simple View of Reading. These findings are discussed below.

The present results suggest that informal home literacy activities are associated with two higher-level comprehension processes, namely knowledge integration and knowledge access. This new finding is important because although a number of studies have shown that informal home literacy activities are associated with preschoolers’ language comprehension and vocabulary knowledge, no study has determined whether home literacy activities are associated with higher-level comprehension processes, some of the most important, if not the most important, components of preschoolers’ language comprehension.

That all said, the composite measure of informal home literacy activities was only related significantly to knowledge integration and knowledge access; the composite measure of informal home literacy activities was not related to text-based processes. Although there are a number of explanations for this finding, one plausible explanation might be that the nature of informal home literacy activities are such that they do not focus on learning the facts of the text itself. Rather, informal home literacy activities focus on the understanding or *comprehending* a text, which requires the comprehender to access their prior knowledge and integrate this prior knowledge with text-based information. Indeed, by definition informal home literacy activities are incidental (Sénéchal, 2006), which means there is no direct focus on the text itself.

The present findings also suggest that there shared variance between informal home literacy activities informal home literacy activities and language comprehension is the same shared variance that is between informal home literacy activities and higher-level comprehension processes. Previous research suggests that informal home literacy activities directly contribute to the language comprehension abilities of kindergarten children (e.g., Sénéchal & LeFevre, 2002). However, this research did not assess higher-level comprehension processes, which are strongly related to language comprehension. The current results of the regression analyses predicting language comprehension not only suggest that informal home literacy activities contribute to both higher-level comprehension processes and language comprehension but they also suggest that these contributions completely overlap. Indeed the results of the regression analyses predicting language comprehension suggest that once the variance attributed to higher-level comprehension processes are partialled out, informal home literacy activities do not account for any unique variance in language comprehension. Thus, it is possible that the contributions of informal home literacy activities to language comprehension is indirect because informal home literacy activities directly influence higher-level comprehension processes rather than language comprehension. That is, informal home literacy activities influence higher-level comprehension processes which in turn, influences language comprehension. In order to test this possibility, future research should use structural equation modeling in order to test for mediating influences.

With respect to theoretical frameworks, the present results provide some evidence that the Home Literacy Model is compatible with the Simple View of Reading. According to the Simple View of Reading, reading is composed of two components: word decoding and comprehension ability. In the context of the Home Literacy Model one would therefore expect formal home literacy activities, which contribute to word skills, to be related to the word decoding component of the Simple View of Reading, and informal home literacy activities, which contribute to vocabulary and language comprehension, to be related to the comprehension component of the Simple View of Reading. Consistent with this expectation, the present study shows that whereas informal home literacy activities are related to language comprehension and specific comprehension abilities, informal home literacy activities are not related to letter-word decoding and phonemic awareness.

Nevertheless, the present study does not test the Simple View of Reading using formal home literacy activities. Consequently, one avenue for future research would be to examine the relationship between the Home Literacy Model and the Simple View of Reading by including measures of both formal and informal home literacy activities. If the Home Literacy Model is truly compatible with the Simple View of Reading than formal home literacy activities should predict performance on measures of word decoding skills but not specific comprehension processes. On the other hand, measures of informal home literacy activities should predict performance on measures of specific comprehension processes but not measures of word decoding.

Besides these new findings, the present results also replicate some important previous findings. For example, the regression analyses showed that higher-level comprehension abilities accounted for 53.7% of the variance in language comprehension; a finding that is consistent with Hannon and Frias’ results (2012). In addition, the present results also showed that informal home literacy activities predict language comprehension but not letter-word decoding and phonemic awareness, a finding that is consistent with a number of previous studies (e.g., Sénéchal, 2006; Sénéchal & LeFevre, 2002; Sénéchal & LeFevre, 2014). Moreover the composite measure home literacy activities correlated with the measure of vocabulary (e.g., Sénéchal & LeFevre, 2002).

Despite the present study’s new insights there are, nevertheless, some limitations. One limitation is that the present study does not establish a causal relationship. For example, question asking could be manipulated in an experiment where one group of preschoolers gets questions and a second group of preschoolers does not get questions. A second limitation is that although the informal home literacy measure querying the number of questions per book asked about the frequency of questions per book, it did not probe the types of questions. For example, did parents ask questions, such as *What will happen next? How does the story relate to our home?* and so on. By understanding the varying contributions that different types of questions make to different specific higher-level comprehension processes parents can then begin using more specific types of questions during storybook reading. Finally, because the present study did not include measures of formal home literacy activities, the relationship between formal home literacy activities and high-level comprehension processes is unknown. Given that previous research suggests that language comprehension is correlated with both formal and informal home literacy activities (e.g., Sénéchal & LeFevre, 2002) and given that specific comprehension processes are highly related to language comprehension (e.g., Hannon & Frias, 2012), one might expect that both formal and informal home literacy activities correlate with specific higher-level comprehension processes.

In summary, this is the first study to show that informal home literacy activities are related to the specific comprehension processes (i.e., knowledge integration, knowledge access) of kindergarten children. Further analysis showed that the shared variance between informal home literacy activities and language comprehension is the same shared variance between informal home literacy activities and higher-level comprehension processes. In other words, the contributions that informal home literacy activities make to language comprehension are not independent of the contributions that informal home literacy activities make to higher-level comprehension processes.

## Figures and Tables

**Table 1.** Sample Paragraph for the Preschooler Component Processes Task.

Animated Introduction	
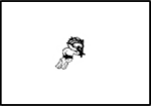	*Mark was looking at sea life while swimming in the ocean. While looking at the sea life, Mark learned:*
Paragraph	
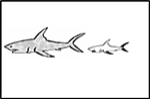	*A LORK looks like a SHARK but a LORK is smaller*.
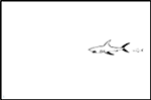	*A PARM looks like a LORK but a PARM is smaller.*
Relationship:	Shark > LORK > PARM
Test Statements	
Text Memory	
A LORK looks like a SHARK.	A LORK does not look like a SHARK.*(n)*
A PARM looks like a LORK.	A PARM does not look like a LORK. *(n)*
A LORK is smaller than a SHARK.	A SHARK is smaller than a LORK. *(n)*
A PARM is smaller than a LORK.	A LORK is smaller than a PARM. *(n)*
Text Inferencing	
A PARM looks like a SHARK.	A PARM does not look like a SHARK. *(n)*
A PARM is smaller than a SHARK.	A SHARK is smaller than a PARM. *(n)*
Low-knowledge integration	
< not possible >	
Medium knowledge integration	
A LORK is smaller than a WHALE.	A WHALE is smaller than a LORK. *(n)*
A PARM is smaller than a WHALE.	A WHALE is smaller than a PARM. *(n)*
High knowledge integration	
Like WHALES, LORKS have fins.	Like TURTLES, LORKS have legs. *(n)*
Like WHALES, PARMS have fins.	Like TURTLES, PARMS have legs. *(n)*
Low knowledge access type II	
A TURTLE is smaller than a SHARK.	A SHARK is smaller than a TURTLE. *(n)*

**Table 2.** Means, Standard Deviations, Ranges, Skew, and Kurtosis for Measures of Home Literacy Activities, Literacy Measures and Parents’ Education Level (*n = 120*).

	M(SD)	Range	Skew	Kurtosis
Measures of Informal Home Literacy Activities				
Composite measure	0.01 (1.54)	−3.05–5.77	0.41	1.44
Number of books per week	4.44 (3.63)	0–21	2.26	0.69
Number of questions perbook	1.81 (1.00)	0–3	−0.71	−0.49
Literacy Measures				
Language comprehension (max=20)	12.42 (3.43)	5–20	0.19	−0.41
Vocabulary knowledge (max = 20)	14.70 (2.60)	6–20	−0.50	0.23
Letter-word decoding (max = 76)	20.73 (9.39)	4–49	0.74	0.14
Phonemic Awareness (max = 32)	5.38(4.53)	0–23	1.54	2.18
Composite higher-level text processing	0.01 (1.80)	−4.68–3.48	−0.19	−0.77
Composite higher-level integration	0.01 (2.43)	−5.96–3.46	−0.58	−0.64
Higher-level knowledge access (max = 4)	3.07(1.03)	0–4	0.94	0.30
Control Variable				
Parents’ education level	5.45 (2.26)	1 – 9	−0.13	−1.07

**Table 3.** Correlations among Measure of Informal Home Literacy Activities and Measures of Literacy (*n = 120*).

		1	2	3	4	5	6	7	8	9
1.	Composite measure home lit activities	---	.20^*^	.21^*^	.09	.09	.14	25^*^	.31^*^	.20^*^
2.	Language comprehension		---	.58^*^	.42^*^	.34^*^	.61^*^	.71^*^	.43^*^	.25^*^
4.	Vocabulary knowledge			---	.43^*^	.33^*^	.47^*^	.45^*^	.35^*^	.17
5.	Letter-word decoding				---	.89^*^	.38^*^	.42^*^	.24^*^	.18^*^
6.	Phonemic awareness					---	.36^*^	.39^*^	.28^*^	.15
7.	Composite high-level text processing						---	.61^*^	.33^*^	.16
8.	Composite high-level knowledge integration							---	.51^*^	.22^*^
9.	High-level knowledge access								---	.18^*^
10.	Parents’ education level									---

Note.

* < .05

**Table 4.** Correlations between the Measure of Informal Home Literacy and Literacy Measures for Kindergarten Children after the Influences of Parental Education have been Removed (*n* = 120).

Composite Measure of Home Literacy	
Language comprehension	.13
Vocabulary	.16
Letter-word decoding	.03
Phonemic awareness	.03
H-level text processing	.10
H-level knowledge integration	.18^*^
H-level knowledge access	.24^*^

Note.

* p < .05.

**Table 5.** Regression Analyses with Combined Home Literacy Activities as First and Last Predictor of Language Comprehension (*n = 120*).

		R^2^	ΔR^2^	F
(i) Composite Measure of Home Literacy Activities First				
1.	Informal home literacy activities^a^	.040	.040	4.93^*^
2.	Combined knowledge integration	.456	.496	105.78^*^
3.	Combined text processing	.546	.050	12.69^*^
4.	Knowledge access	.550	.006	1.23
(ii) Composite Measure of Informal Home Literacy Activities Last				
1.	Combined knowledge integration	.495	.495	115.72^*^
2.	Combined text processing	.545	.050	12.75^*^
3.	Knowledge access	.550	.005	1.42
4.	Informal home literacy activities^a^	.550	.000	0.22

Note.

* p < .05.

a This is the composite measure of informal home literacy activities.

## Footnotes

Note 1: The present study follows the tenets of many taxonomies of language comprehension that classify word identification and phonemic awareness processes as lower-level because they focus on letters/individual words and knowledge integration and inferencing processes as higher-level because they aid in the construction of meaning-based text representations.

Note 2: As noted in Hannon and Frias (2012, p. 897), “the literature includes many terms to describe language comprehension ability, such as oral language skills, oral comprehension, reading comprehension, and listening comprehension.” Following Hannon and Frias, the present study “uses the terms *reading comprehension ability* and *reading skills* to describe abilities/skills that are exclusively reading and the terms *language comprehension ability* and *oral language skills* to describe abilities/skills that are either exclusively or primarily oral” (p. 897). For instance, the present study’s measure of language comprehension is identified as a measure of language comprehension because the passages and questions are present aurally and only the answer choices are pictures (Hannon & Frias, 2012).
